# Improvement of Interfacial Adhesion by Bio-Inspired Catechol-Functionalized Soy Protein with Versatile Reactivity: Preparation of Fully Utilizable Soy-Based Film

**DOI:** 10.3390/polym9030095

**Published:** 2017-03-07

**Authors:** Zhong Wang, Haijiao Kang, Wei Zhang, Shifeng Zhang, Jianzhang Li

**Affiliations:** 1 MOE Key Laboratory of Wooden Material Science and Application, Beijing Forestry University, Beijing 100083, China; wangzhong@bjfu.edu.cn (Z.W.); khj0339@bjfu.edu.cn (H.K.); zhangweishe@126.com (W.Z.); 2 Beijing Key Laboratory of Wood Science and Engineering, Beijing Forestry University, Beijing 100083, China

**Keywords:** mussel-inspired, catechol functionalization, interfacial adhesion, full utilization, soy based films, mechanical properties

## Abstract

The development of materials based on renewable resources with enhanced mechanical and physicochemical properties is hampered by the abundance of hydrophilic groups because of their structural instability. Bio-inspired from the strong adhesion ability of mussel proteins, renewable and robust soy-based composite films were fabricated from two soybean-derived industrial materials: soluble soybean polysaccharide (SSPS) and catechol-functionalized soy protein isolate (SPI-CH). The conjugation of SPI with multiple catechol moieties as a versatile adhesive component for SSPS matrix efficiently improved the interfacial adhesion between each segment of biopolymer. The biomimetic adherent catechol moieties were successfully bonded in the polymeric network based on catechol crosslinking chemistry through simple oxidative coupling and/or coordinative interaction. A combination of H-bonding, strong adhesion between the SPI-CH conjugation and SSPS matrix resulted in remarkable enhancements for mechanical properties. It was found that the tensile strength and Young’s modulus was improved from 2.80 and 17.24 MPa of unmodified SP film to 4.04 and 97.22 MPa of modified one, respectively. More importantly, the resultant films exhibited favorable water resistance and gas (water vapor) barrier performances. The results suggested that the promising way improved the phase adhesion of graft copolymers using catechol-functionalized polymers as versatile adhesive components.

## 1. Introduction

Biobased polymers with low costs and density, excellent biodegradability, and acceptable specific properties (gas and oil barrier) are of great importance to replace conventional petroleum-based synthetic materials, which has drawn considerable interest from researchers in recent decades [1,2]. Generally, various biopolymers from different biorenewable resources have been used to develop biobased materials, including proteins, polysaccharides, oils and lipids, and biogenic polyesters [3,4]. Among these biopolymers, soy protein isolate (SPI) derived from the most abundant plant proteins are considered ideal candidates for fabricating eco-friendly biomaterials owing to its superiorities of low cost, biocompatibility, functional side chains, and easily processing properties [5,6]. When the above advantages are taken into account, SPI based films have found a huge potential for utilization in several fields, such as packaging and coatings [3], drug delivery [7], tissue engineering [8], as well as in promising materials for air filtration and electronic devices application [9]. More practical applications have taken advantage of the inherent properties of SPI with multi-functionality, which has been widely developed upon modification for use as a promising active packaging systems over the last decade [2,6]. In particular, it was mostly reported that SPI based films generally exhibit impressive gas and mechanical barrier properties compared to polysaccharides and lipids [5]. However, because of the hydrophilic nature and strong molecular interactions, SPI films are highly sensitive to moisture, and their tensile properties cannot warrant their practical applications.

Therefore, much attention has been dedicated to improving the inherent properties of the SPI based films by various physical or chemical methods. Tremendous efforts in this area could be classified into three categories: (1) physical modification, mainly including physical denaturing treatments (e.g., heating, thermoplastic processing, and ultrasound) and combining it with organic or inorganic filler materials such as natural biopolymers, nanoclay/nanofibrils [10,11]; (2) chemical cross-linking modification, to construct intra- or inter-penetrating network-like systems in SPI matrices using a serials of crosslinkers including aldehydes, epoxy compounds, natural polyphenol, and synthetic resin [12,13]; (3) biochemical techniques, to improve the surface properties of SPI by controlled enzymatic hydrolysis [6]. Among these, the chemical copolymerization by esterification, amidation, and polymer grafting have been proposed as an excellent technique. For instance, Chen et al. reported that the grafting of diethoxy phosphoryl groups on SPI chains resulted in SPI films with robust mechanical properties [14]. Salama et al. prepared a bioactive material using cellulose grafted SPI based on biomimetic mineralization [15]. Given their molecular properties of graft segments, the incompatibility and poor adhesion between the graft segments and the SPI chains often makes a great difference in the performance of SPI-based composites [5,16]. Hence, it is challengeable to exploit novel SPI based graft copolymers that are capable of both robust binding and compatible interactions with polymer matrices.

Inspired by the bioadhesive capability of mussel adhesive proteins (MAPs), considerable efforts have been devoted into designing new bioadhesive polymer materials for biomimetic mussel adhesion [17,18]. As a key component of 3,4-dihydroxyphenylalanine (DOPA), the ortho-dihydroxyphenyl (catechol) moiety can form strong covalent and noncovalent linkages with various substrate surfaces through H-bonding, metal-ligand complexes, and secondary reactions (e.g., Schiff base reactions or Michael addition) with amine-/thiol-terminated molecules [19]. Bio-inspired from the MAPs mechanism, the co-existence of multiple interactions in the polymeric network might allow us to prepare robust biopolymer films by both noncovalent and covalent bonds, which can overcome the drawbacks of conventional SPI-based graft copolymers. For example, Li et al. have synthesized and characterized a strong, water-resistant wood adhesive based on soy protein by introducing a key functional dopamine group [20]. More recently, we reported that the integration of catechol functionalized microcrystalline celluloses or nanoclay into the SPI matrices led to a significant increase of the mechanical behavior and water resistance [21,22]. In consequence, it is reasonable to believe that the catechol groups conjugated in the SPI backbone with versatile reactivity and good compatibility could offer effective adhesion strength with biopolymer matrices.

Because of the intrinsic carbohydrate structure, soluble soybean polysaccharide (SSPS) is also derived from soybean crops, issues such as naturally solubility and poor mechanical properties are often still limiting their applications in packing materials [23,24]. Studies have demonstrated that the introduction of oxidized sugars as a natural crosslinker into the SPI matrix will promote the remarkable cross-linking in the soy flour based resin [25]. Taking cues from adhesion proteins, it was hypothesized that abundant primary amines in the SPI as well as hydroxyl groups in SSPS chains could be multiple cross-linked by conjugated catechol groups to form a robust soy-based composites.

**Scheme 1:**
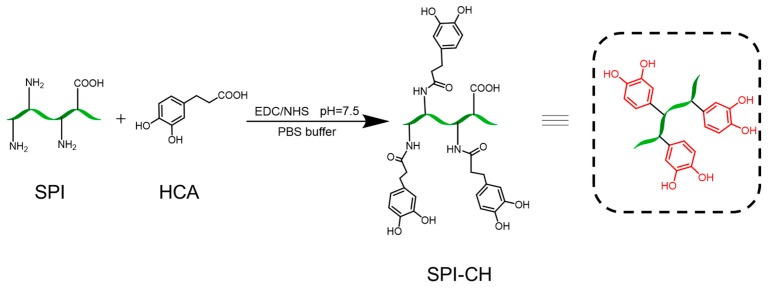
Synthesis of catechol-functionalized soy protein isolate (SPI-CH) conjugates by 1-ethyl-3-(3-dimethylamino-propyl)-carbodiimide hydrochloride (EDC) coupling agent using standard carbodiimide chemistry.

**Scheme 2:**
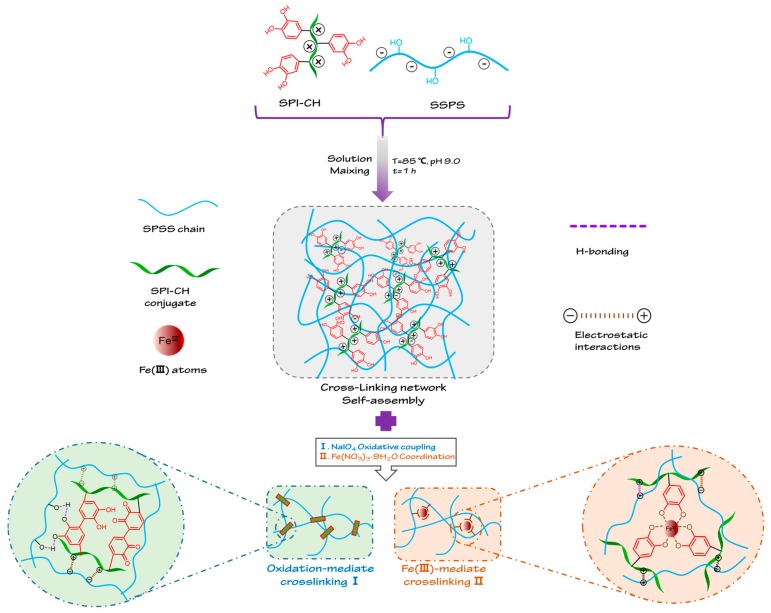
Schematic representation of preparation and crosslinking formation of soy-based composite films.

Herein, the primary objective of this work was to develop fully renewable and robust soy-based composite film using catechol-functionalized SPI (SPI-CH) and SSPS via a biologically inspired strategy. Firstly, mussel-inspired SPI-CH hybrid conjugation was synthesized based on carbodiimide chemistry (Scheme 1), and reactive catechol groups conjugated in the SPI backbone were integrated into the SSPS matrix. In this film system, a biocompatible SPI was selected as a hydrophilic polypeptide skeleton to provide primary amine groups, and catechol groups as a crosslinker to crosslink the SPI and SSPS backbone by an oxidizing/chelating process (Scheme 2). Owing to the existence of multiple physicochemical interactions (including H-bonding and covalent and/or coordination bonds) from the polymeric network in the films, the soy-based films exhibited significant enhancements in mechanical and water resistance properties. Furthermore, the effect of the type of SPI-CH crosslinking on the microstructure, thermostability, surface hydrophilicity of the films was investigated.

## 2. Materials and Methods

### 2.1. Materials

Soy protein isolate (SPI) powder (95% protein content) was purchased from Yuwang Ecological Food Industry Co., Ltd. (Shandong, China) and SSPS was provided by Fuji Oil (Osaka, Japan). 3,4-dihydroxy hydrocinnamic acid (HCA, 98% pure), 1-ethyl-3-(3-dimethylamino-propyl)-carbodiimide hydrochloride (EDC), *N*-hydroxysuccinimide (NHS), and Fe(NO_3_)_3_·9H_2_O (99.9%) were purchased from Tianjin Heowns Biochem Co., Ltd., Tianjin, China. NaIO_4_ (99.9% pure) and other chemicals were purchased from Beijing Chemical Reagents Co., Ltd., Beijing, China, and used as-received without further purification. Distilled (DI) water was used in all aqueous solution preparations.

### 2.2. Synthesis of Catechol-Conjugated SPI (SPI-CH)

SPI-catechol conjugate was synthesized with slight modification through the carbodiimide catalyzation method: Briefly, SPI (10.0 g) was dissolved in DI water (200 mL) and stirred uniformly, and the pH of the dispersion was adjusted to 8.5 with NaOH solution (1M). HCA (4.2 g) and EDC (5.4 g) were dissolved in DI water/ethanol (100 mL, 1:1 *v*/*v*) and NHS (3.0 g) was added to the mixture to activate the carboxylic group on HCA molecules under cooling in an ice bath. After 30 min, the activated HCA solution was added dropwise to the SPI solution and the pH value of the reaction solution was maintained at 8.5 over a 24-h reaction in an ambient atmosphere. The reaction solution was dialyzed (MWCO: 8000–10,000, SpectraPor, Beckman Instruments, Mountainside, NJ, USA) thoroughly against NaOH solution (pH 8.0–8.5) for 48 h and DI water for 12 h, and then lyophilized to obtain the SPI-CH conjugate product.

### 2.3. Preparation of Soy-Based Composite Films with SPI-CH

The SSPS based films were prepared freshly with SSPS and SPI-CH conjugates using a casting method. For the control film, SSPS (5 g) and glycerol (2.5 g) were dispersed in DI water and heated in a water bath at 85 °C for 1 h under vigorous stirring. To fabricate SPI-CH crosslinked SPSS films, a predetermined amount of SPI-CH, Fe(NO_3_)_3_·9H_2_O or NaIO_4_ (as listed in Table 1) was dispersed in the SPSS (5 wt %) solution with the aid of plasticizer glycerol (50% based on the weight of SSPS) and constantly stirred for 1 h at 85 °C. The mixture was then ultrasound-treated (VC-750, Sonics and Materials, Danbury, CT, USA, 750 W) for 15 min and poured into Teflon-coated plates, then vacuum-dried at 45 °C for 24 h.

### 2.4. Chemical Characterization of SPI-CH

The synthesis of SPI-CH was confirmed by nuclear resonance (^1^H NMR, JEOL ECS, Tokyo, Japan, 400 MHz) spectra and UV-Vis spectrophotometry (TU-1901, Purkinje General Instrument Co., Ltd., Beijing, China). The HCA, SPI, and SPI-CH samples were dried and dissolved into DMSO-*d*_6_. Attenuated total reflectance-Fourier transform infrared (ATR-FTIR) spectra of SPI-CH conjugates was recorded using a Nicolet Nexus 6700 spectrometer (Thermo Scientific, Madison, WI, USA) from 4000 to 650 cm^−1^ wavenumbers with 32 scans.

### 2.5. Film Characterization

#### 2.5.1. Preconditioning

In order to avoid the influences of moisture content and post cross-linking on the protein based films properties [26], all the films were equilibrated in a conditioning room (25 ± 2 °C and 50% ± 2% relative humidity (RH)) for 48 h before further characterization. Meanwhile, the results of each measurement characterization were measured at the same time.

#### 2.5.2. Structural Characterization

ATR-FTIR analyses were conducted to illustrate the chemical structural differences of the control film and crosslinked films as described previously [22]. X-ray diffraction (XRD) patterns were observed on a D8 Advance diffractometer (Bruker AXS, Karlsruhe, Germany) equipped with a Cu-K radiation source (*k* = 1.54060 Å) at 40 kV with a current of 40 mA. The relative intensity was gathered from 5° to 60° with a speed of 2° min^−1^. The fracture surface morphologies of the films were examined using a field-emission scanning electron microscope (FESEM, Hitachi SU8010, Tokyo, Japan) after coating the samples with gold. A thermogravimetric analyzer (TGA Q50: TA instruments, New Castle, DE, USA) was used to investigate the thermal stability and degradation behavior of the SSPS films before and after crosslinking. Film samples were scanned from 25 to 600 °C at a heating rate of 10 °C·min^−1^ under a constant nitrogen atmosphere (100 mL·min^−1^).

#### 2.5.3. Surface Contact Angles

The surface hydrophobicity of the soy-based films was investigated with a OCA-20 contact angle goniometer (CA, DataPhysics Instruments GmbH, Filderstadt, Germany) via the sessile droplet method with 3 μL of distilled water as described previously [13]. The angles of both sides were recorded at intervals of 0.1 s for 180 s. The contact angles data for each sample were averaged over at least five points.

#### 2.5.4. Water Vapor Permeability

The water vapor permeability (WVP) were determined by use of a W3/031 permeability analyzer (Labthink Instrument, Jinan, China) according to the ASTM E 96 standard method. Five specimens with a surface area of 8 cm^2^ for each film were tested at 23 °C and 50% RH using silica-gel desiccant. The amount of water vapor penetrated through the film was determined by measuring the weight increase of the specimens. The WVP was calculated based on the report by Li et al. [27].

#### 2.5.5. Water Solubility Testing

The total soluble matter (TSM) of the soy-based films was evaluated using the same procedure described by Kang et al. [21]. Briefly, the specimens (20 × 20 mm^2^) were desiccated overnight in an air-circulating oven at 103 °C and weighed to determine their dry mass (*m*_i_). Specimens were then immersed in 30 mL of water (pH = 7) containing traces of sodium azide (0.02%, *w*/*v*) for microorganism prevention at 25 °C. After a 24 h water submersion, the specimens were dried to a final constant weight (*m*_t_). Five replicates for each test was carried out. The total soluble matter (TSM) of the films was calculated as follows:

$$\mathrm{TSM}\;\left(\%\right)=\left(m_{i}-m_{t}\right)/m_{i}\times 100$$

#### 2.5.6. Mechanical Behavior Analysis

The mechanical properties of the resultant soy-based films, including tensile strength (TS), elongation at break (EB), and Young’s modulus (YM), were determined with a universal material testing machine (INSTRON 3365, Norwood, MA, USA), at a loading speed of 50 mm·min^−1^ and a gauge length of 50 mm. At least five specimens (10 × 80 mm^2^) of each film were tested to obtain the average mechanical tensile data. The thickness was measured with a digimatic micrometer (Measuring&Cutting Tool Works Co., Ltd., Shanghai, China) (0–25 ± 0.001 mm) and the average values of three replicates were reported.

### 2.6. Statistical Analysis

The Duncan’s multiple-range test was performed to statistically compare the mean difference among the film specimens at *p* = 0.05. Experimental treatments were conducted using at least five replicates using the analysis of variance (ANOVA, SPSS 9.0.1, SPSS Inc, Chicago, IL, USA) [13].

## 3. Results and Discussion

### 3.1. Synthesis and Characterization of Catechol-Conjugated SPI

**Figure 1:**
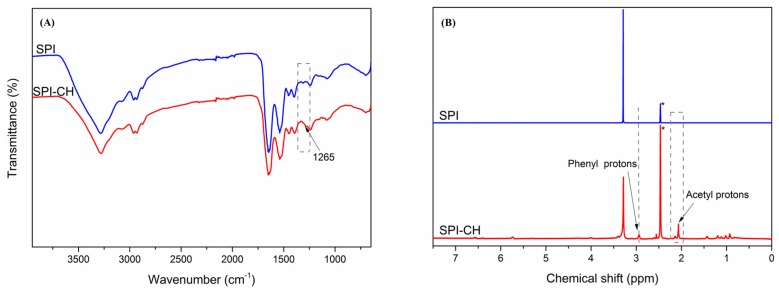
(**A**) FT-IR spectra SPI and SPI-CH powder; (**B**) 1H NMR spectrum of SPI and SPI-CH in DMSO.

The catechol-functionalized SPI conjugation was synthesized by the conjugation of hydrocaffeic acid to the SPI backbone via carbodiimide coupling protocols (Scheme 1). The covalent conjugation of the catechol molecules on the SPI backbone was verified using ^1^H NMR and FT-IR spectra (Figure 1). The FT-IR spectrum of SPI-CH showed a single characteristic absorption band at 1265 cm^−1^ attributed to the stretching vibration of phenolic alcohol bands [28]. The ^1^H NMR spectra conjugates were gathered to further probe the nature of the catechol functional SPI. As shown in Figure 1B, there is a noticeable peak of the acetyl protons between 2.1 and 2.36 ppm in the SPI-CH spectrum [29]. The peak at 2.75 ppm can be attributed to phenyl group protons in the hydrocaffeic acid [30]. These changes in FT-IR and ^1^H NMR spectra signal the successful conjugation of catechol groups onto the SPI backbones. The catechol group substitution rate in the SPI-CH conjugate was 21%, as-determined by the modified Aasheesh’s method [31].

### 3.2. Structural Analysis of Films Crosslinked under Different Conditions

**Figure 2:**
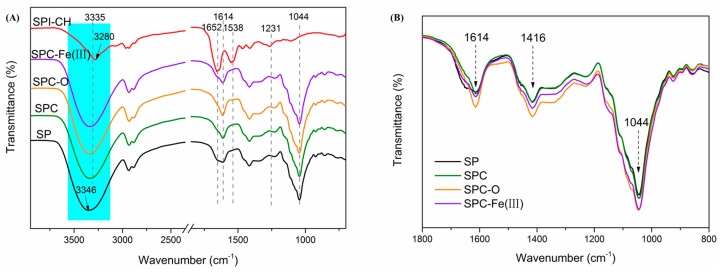
ATR-FTIR spectra of the SPI-CH conjugates, soy-based composite films under different cross-linking conditions in (**A**) full wave number ranges; (**B**) low wavenumber region of 1800–800 cm^−1^.

FT-IR spectroscopy was applied here to investigate the chemical structure of our soy-based composite film samples. Figure 2A shows the ATR-FTIR spectra of the soy-based films and the SPI-CH powder. For the SPI-CH conjugates, the typical amide bands at 1652, 1538, and 1231 cm^−1^ were assigned to amide I (C=O stretching), amide II (N–H bending), and amide III (C–H and N–H stretching), respectively [13,32]. The primary characteristic peaks of the control (unmodified SP film) were 1614 cm^−1^ (amide I, C=O stretching) caused by the existence of protein portion, and 1044 cm^−1^, derived from the rhamnogalacturonan moiety [33]. The broad and strong absorption bands at 3280 and 3346 cm^−1^ are typically attributed to the O–H and N–H bending vibrations of the SPI-CH and the O–H symmetrical stretching vibration of SP, respectively [33,34]. The IR spectra provided direct and powerful evidence of intra- or intermolecular H-bonding interactions according to the change in the O–H vibrational frequency [35,36]. Interestingly, compared to the control, the O–H stretching peaks of the soy-based composite films shifted to a lower wavenumber (3335 cm^−1^) with the addition of the SPI-CH conjugate. Accordingly, these prominent shifts of the O–H absorption bands to lower wavenumbers, are clearly indicating the formation of stronger intermolecular H-bonding interactions between the SPI-CH conjugate and SPSS chains. Chen et al. reported similar observations [37].

To further investigate the H-bonding interactions among SPI-CH and SP chains under different cross-linking conditions, amplification FT-IR spectra (1800 to 800 cm^−1^) were gathered as shown in Figure 2B. After the addition of the SPI-CH conjugates, the absorption band intensities at 1614 and 1044 cm^−1^ increased slightly, indicating that the SPI-CH chain segment was engaged in protein via physicochemical crosslinking [13]. Compared to the SPC film, the increase in absorption peak intensity in the SPC-O and SPC-Fe(III) films was more distinct; this was likely due to the oxidative coupling and multivalent coordination of catechol and/or quinone groups in SPI-CH with the unique crosslinked network structure [28,38]. The FT-IR results helped us to determine that the crosslinked soy-based films composed of SSPS and SPI-CH were formed via effective H-bonding interactions, as opposed to the control.

**Figure 3:**
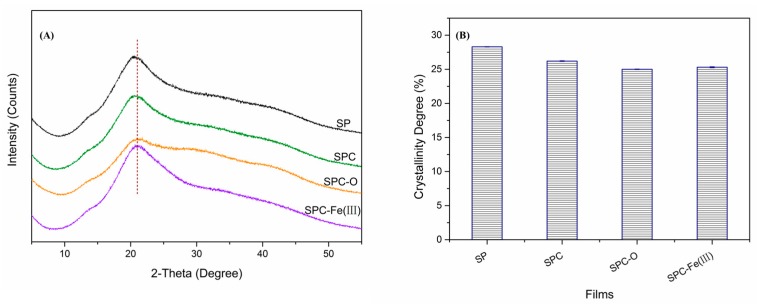
(**A**) X-ray diffraction patterns; (**B**) crystallinity of control and soy-based composite films.

The effects of the SPI-CH conjugates on the composite films’ crystallinity structures were observed by XRD, as shown in Figure 3A. The broad characteristic peak at approximately 2θ = 22.5° reflected typical SSPS crystal conformation [23]. The XRD pattern of SPI did not exhibit obvious sharp peaks of crystalline structures, indicating a favorable chemical compatibility and physical distribution of the SPI-CH conjugates in the resultant composites [27]. For the unmodified and/or crosslinked SPC films, the typical peaks of SSPS crystallites shifted to around 23.2° and decreased in intensity after NaIO_4_ or Fe(III) ion incorporation mainly due to the inhibiting effects on the formation of SSPS crystallites by multiple interactions between the SPI-CH and SSPS matrix. Similar conclusions were drawn from the calculated degrees of crystallinity of the soy-based composites. We found that the SPC film crystallinity was lower than that of the control when crosslinking with NaIO_4_ or Fe(III) ions (Figure 3B). This likely resulted from the physicochemical crosslinking network between the amphipathic SPI-CH chain segment and SSPS matrix in accordance with the FT-IR analysis [12,39]; taken together, the robust crosslinking network was formed in the composite samples due to the strong intra- or intermolecular H-bonding and multiple physicochemical bonding.

### 3.3. Micromorphology of Soy-Based Composite Films

**Figure 4:**
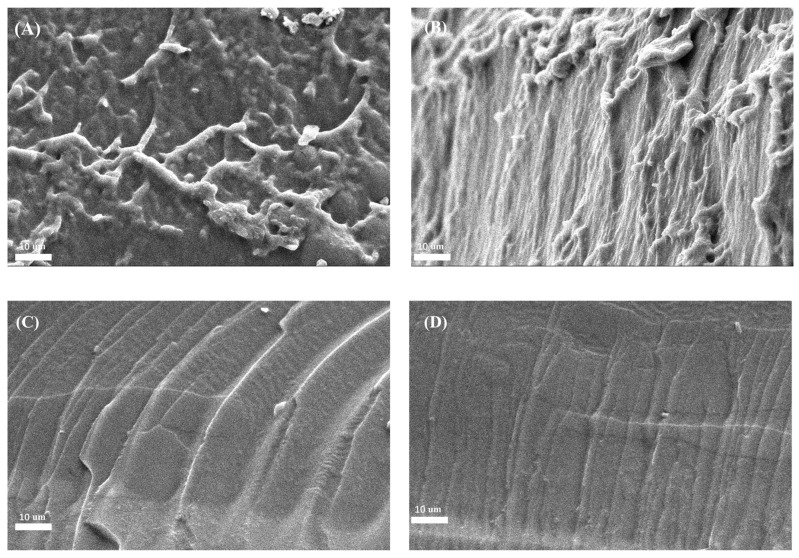
FE-SEM images of the fracture surfaces of (**A**) unmodified SSPS; (**B**) SPC; (**C**) SPC-O; (**D**) SPC-Fe(III) composite films.

Typical scanning electron microscopy (FE-SEM) images revealed the fracture surface morphologies of the soy-based films (Figure 4). The control film showed a discontinuous and coarse fractured surface. Compared to the control, the SPC composite displayed a relatively irregular and rough fractured morphology with clear interlocking and entanglement effects [39], which indicated the changes of the soy-based composite microstructure (Figure 4B). These effects also implied that catechol-functionalized SPI promoted the adhesion and compatibility of the SPI molecules in the composite because of its unique adhesive interface with physicochemical effects. With the addition of NaIO_4_ or Fe(III) ions, however, the micrograph became significantly smooth and dense while the ridge-and-valley pattern of the control disappeared, thus evidently increasing the films’ tensile strength (Figure 4C,D). This can be attributed to the further enhancement in interfacial adhesion properties between the SPI-CH and the SSPS matrix based on multiple crosslinking processes; FT-IR and XRD analysis results support this conclusion.

### 3.4. Mechanical Properties of Soy-Based Composite Films

**Figure 5:**
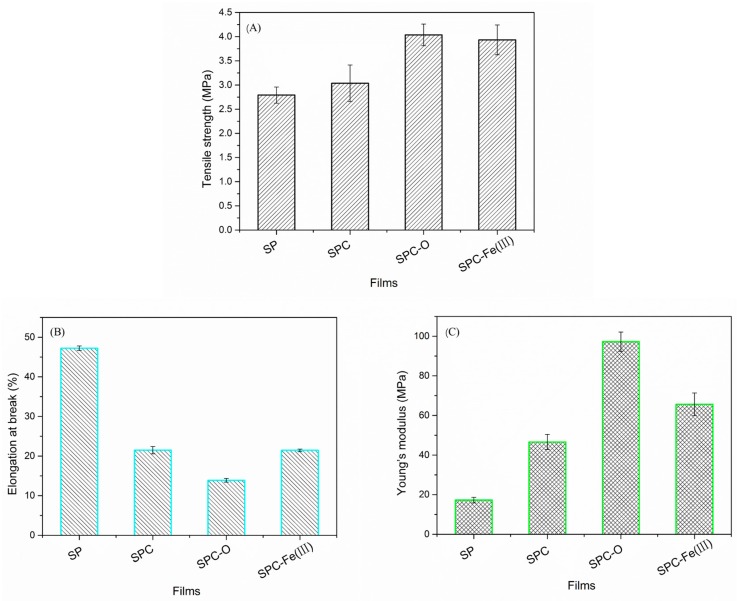
Tensile properties (**A**) TS; (**B**) EB; and (**C**) YM of control and soy-based composite films.

Generally, direct blending of natural matrices and biopolymers improves their tensile stress and modulus due to internal blend miscibility and filling effects [40]. However, the relatively weak phase interfacial adhesion is inferior to the diphase bonding between matrices and reinforced phases [41]. The effect of catechol-functionalized SPI on the mechanical properties (including TS, EB, and YM) of the composite films by controlling the crosslinking conditions was investigated, and is depicted in Figure 5. The detailed data is listed in Table 2. The thickness of soy-based films ranged from 0.205 to 0.245 mm (as shown in Table 2). The control film showed a TS of 2.80 MPa, YM of ~17.24 MPa, and stretches to ~47.2% elongation before failure, in accordance with previous reports [23]. Integration of SPI-CH into the SSPS matrix led to a slight increase in TS, as confirmed by the statistical analysis (*p* > 0.05). YM of the SPC film increased to 46.60 ± 3.81 MPa, counting a 170.3% increase (*p* < 0.01). Such significant improvement in the SPC composite can be attributed to the strong cohesion force between the SSPS and SPI-CH segments with effective physicochemical interactions [37]. The mechanical properties (TS and YM) increased significantly (*p* < 0.01) after introducing the crosslinking network: In the catechol-Fe(III) coordination crosslinking process, TS and YM increased to 3.93 MPa (an increase of 40.9%) and 65.61 MPa (an increase of 280.6%), compared to the control, respectively. The YM of the oxidatively coupled crosslinked composite film was the highest (97.22 MPa) out of all samples, and counted more than five times greater than that of the unmodified SP film (*p* < 0.01). The crosslinking process created a robust network that allowed the composites to bear stronger force and efficiently transfer stress in the interfacial region, leading to their high modulus and tensile strengths; these findings are supported by the FE-SEM morphologies described previously.

Taken together, these results demonstrate that catechol-functionalized SPI has versatile active binding sites and the ability to form a robust crosslinking network by multiple physicochemical interactions (e.g., H-bonding and covalent and/or coordination bonds). The SPI-CH conjugate can act not only as natural reinforcement but also participate in various crosslinking reactions (see FT-IR, XRD analyses) due to high interfacial adhesion and biocompatibility with the soy-based matrix.

### 3.5. Thermal Behavior of Soy-Based Composite Films

**Figure 6:**
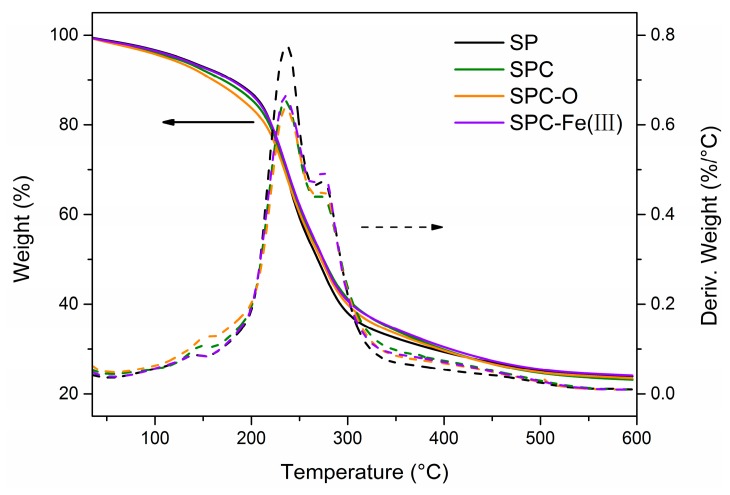
Thermogravimetric (TG) and derivative thermogravimetric (DTG) curves of control and soy-based composite films.

The thermal properties of the soy-based composite films were examined by TGA as shown in Figure 6. The thermo-degradation data is presented in Table 3. The soy-based composites exhibited a similar pattern of thermal behavior relative to the control film, and underwent two degradation stages: One typical, from 20 to 120 °C for the dehydration process, and another ranking from 120 to 450 °C mainly for glycerol, main backbone polysaccharide, and/or peptide decomposition [10]. Compared to the generally accepted thermo-degradation mechanism of SPI, it was found that the typical degradation peak of SPI (around 310 °C) disappeared in the composite films, suggesting a favorable combination and compatibility of biphase composites [21]. The degradation peaks in the second stage (around 235 °C) of composites significantly decreased in comparison to the control, which likely implies the enhanced thermal stability in the composite films due to the multiple physicochemical bonding between the SPI-CH conjugates and the soy-based matrix [22]. In addition, as the composite films further crosslinked, higher residual masses at 550 °C were observed in the SPC-O and SPC-Fe(III) films (Table 3); the robust network was enhanced via multiple interactions in the resultant composites, as confirmed by the FT-IR and XRD results. In conclusion, the addition of catechol-functionalized SPI facilitates slight improvement to the thermal stability of SPI-based films.

### 3.6. Water Resistance and Surface Hydrophobicity of Soy-Based Composite Films

Water resistance is one of the most important properties of bio-based films in terms of practical application due to the matrix’s inherent hydrophilicity [3]. The water resistance (TSM), surface hydrophobicity (WCAs), and water vapor permeability (WVP) results are summarized in Table 4. The addition of SPI-CH decreased TSM by 19.2% compared to the control (*p* < 0.05), and the further crosslinking of SPI-CH contributed to further enhancement in water resistance and a slight decline in TSM from 74.63% (SPC film) to 62.14% (SPC-O film), and 67.82% (SPC-Fe(III) film), respectively (*p* > 0.05). Compared to the unmodified SP film, there was a 48.6% decrease (*p* < 0.01) in TSM when SPI-CH was oxidatively coupled, which were much lower than these of reported SSPS film by Mortazavi et al [23]. This is primarily ascribed to an active adhesive interface in the composites, which promoted polysaccharide and peptide chain crosslinking and formed a denser polymeric network structure via multiple interactions, resulting in an effective resistance to the permeation of water molecules [13]. The WVP values of the film samples (Table 4) after the SPI-CH incorporation with different crosslinking processes also supports these observations. WVP was significantly different (*p* < 0.01) between the SP and SPC-O and/or SPC-Fe(III) films. The WVP values of all soy-based composite films were lower than the control one, which was consistent with the fact of better barrier properties [1]. It was indicated that the soy-based composite films prepared here could be readily applied in food packaging because of their efficient barrier properties.

Crosslinking also may affect the films’ surface hydrophobicity due to multiple interactions in the polymeric network, as well as decreased polar groups in the film’s surface [39]. As shown in Table 4, the unmodified film had a WCAs of 25.9°, which increased to 44.7° after the incorporation of SPI-CH (*p* < 0.05); and increased further (55.8°) after the catechol-Fe(III) coordination crosslinking (SPC-Fe(III) film) (*p* < 0.05). Compared to that of reported SPI or SSPS film, it was found that integration of cross-linked SPI-CH system into the SSPS matrix results in notable improvement of the surface hydrophilicity of composite films. This observation combined with the FT-IR, XRD, and FE-SEM results indicated that multiple physicochemical interactions resulted in the re-arrangement of polar/nonpolar groups under the dense crosslinking network structure (–OH, –NH_2_, –COOH, and –SH), which improved the surface hydrophobicity of the composite films [22].

## 4. Conclusions

In this study, the fully soy-derived, highly biocompatible, and bio-inspired soy-based composite films based on catechol-conjugated SPI and SSPS were developed. The polymer with multiple catechol groups apparently generated multiple physicochemical interactions (including H-bonding and covalent and/or coordination bonds) with the SSPS matrix, which improved the bond strength of SPI chains to the composite film. The catechol-conjugated SPI was confirmed by the ^1^H NMR and FT-IR analysis with the EDC/NHS chemistry. The formation of a robust crosslinking network through oxidative coupling and catechol-Fe(III) coordination was confirmed by ATR-FTIR, XRD, FE-SEM, and TGA analyses. Under these multiple interactions, the composites exhibited significant enhancement of mechanical properties and water resistance compared to the control. With the addition of 8 wt % SPI-CH via oxidative coupling, TS and YM of the composite films increased by 44.6% and 463.9%, respectively, while TSM decreased by 32.7%, compared to the control. Besides, the moderate water vapor permeability and thermal stability of the soy-based films fabricated in this study could meet the requirements of certain applications.

This work provides a bio-inspired methodology for the efficient and economic utilization of soybean food industrial products. The films fabricated here may represent an approach for producing eco-friendly, soy-based, all components bio-derived composite materials.

## Figures and Tables

**Table 1:** Feed composition and summary of soy-based composite films.

Codes	SSPS (g)	Glycerol (g)	Water (g)	SPI-CH (g)	NaIO_4_ (g)	Fe(III) (g, 4%)
SP	5	2.5	95	-	-	-
SPC	5	2.5	95	0.4	-	-
SPC-O	5	2.5	95	0.4	0.005	-
SPC-Fe(III)	5	2.5	95	0.4	-	0.015

**Table 2:** Tensile properties (tensile strength (TS), elongation at break (EB), Young’s modulus (YM)) of control and soy-based composite film samples.

Film Code	Thickness	Tensile strength	Elongation at break	Young’s modulus
	(mm)	(MPa)	(%)	(MPa)
SP	0.233 (0.031) ^a^	2.80 (0.17)	47.2 (0.48)	17.24 (1.41)
SPC	0.245 (0.024)	3.04 (0.38)	21.5 (0.93)	46.60 (3.81)
SPC-O	0.205 (0.009)	4.04 (0.22)	13.9 (0.47)	97.22 (4.89)
SPC-Fe(III)	0.217 (0.010)	3.93 (0.31)	21.4 (0.33)	65.61 (5.71)

^a^ Mean (standard deviation).

**Table 3:** Thermo-degradation data of control and soy-based composite film samples.

Curve	SP	SPC	SPC-O	SPC-Fe(III)
T_i_^a^ (°C)	214.51	213.70	215.16	213.25
T_max_^b^ (°C)	237.15	235.49	235.90	235.12
Residual mass (wt %) at 550 °C	27.76	27.54	28.18	28.08

^a^ Initial temperature of degradation; ^b^ Temperature of maximum degradation rate.

**Table 4:** Total soluble matter (TSM), water vapor permeability (WVP), and surface hydrophobicity (WCAs) of control and soy-based composite film samples.

Film code	Total soluble matter	Water vapor permeability	Water contact angles
	(%)	(×10^−12^ g·(cm·s·Pa)^−1^)	(°)
SP	92.35 (0.85) ^a^	9.83 (0.23)	25.9 (3.8)
SPC	74.63 (0.97)	8.10 (0.31)	44.7 (2.1)
SPC-O	62.14 (1.04)	6.74 (0.19)	37.5 (1.3)
SPC-Fe(III)	67.82 (0.65)	7.32 (0.34)	55.8 (0.9)

^a^ Mean (standard deviation).
